# Alterations in hippocampal somatostatin interneurons, GABAergic metabolism, and ASL perfusion in an aged male mouse model of POCD aggravated by sleep fragmentation

**DOI:** 10.14814/phy2.70153

**Published:** 2024-12-08

**Authors:** Yun Li, Jiafeng Yu, Ningzhi Yang, Siwen Long, Yize Li, Lina Zhao, Yonghao Yu

**Affiliations:** ^1^ Department of Anesthesiology Tianjin Medical University General Hospital Tianjin China; ^2^ Tianjin Research Institute of Anesthesiology Tianjin China; ^3^ Department of Critical Care Medicine Tianjin Medical University General Hospital Tianjin China

**Keywords:** aging, hippocampus, magnetic resonance imaging, POCD, sleep fragmentation, somatostatin

## Abstract

Sleep fragmentation (SF) is increasingly recognized as a contributing factor to postoperative cognitive dysfunction (POCD). Given the critical roles of somatostatin (SST) interneurons, associated gamma‐aminobutyric acid (GABA)ergic neurotransmitters, and hippocampal perfusion in sleep‐related cognition, this study examined changes in these mechanisms in preoperative SF affecting POCD induced by anesthesia/surgery in aged male mice. The Morris water maze (MWM), novel object recognition (NOR), and Y maze tests were utilized to evaluate POCD. Arterial spin labeling (ASL) was employed to measure hippocampal regional cerebral blood flow (rCBF). In vitro assays quantified the levels of GABAergic metabolites—such as SST, neuropeptide Y (NPY), glutamic acid decarboxylase 1 (GAD1), vesicular GABA transporter (VGAT), and GABA and the distribution of SST interneurons in the hippocampus through enzyme‐linked immunosorbent assay and immunofluorescence. Preoperative 24‐h SF exacerbated anesthesia/surgery‐induced spatial memory impairments observed in the MWM, NOR, and Y maze tests. Preoperative 24‐h SF significantly increased the number of SST interneurons in hippocampal CA1, elevated hippocampal levels of SST, NPY, GAD1, and GABA, and reduced the rCBF. Preoperative SF aggravated POCD in aged male mice, with an increased number of SST interneurons in hippocampal CA1, elevated hippocampal GABAergic metabolites, and a further reduction in rCBF.

## Graphical abstract

Preoperative 24‐h sleep fragmentation aggravates anesthesia/surgery‐induced POCD in aged mice, accompanied by an increase in the number of SST interneurons in the hippocampal CA1 region, an increase in the levels of hippocampal SST, NPY, GAD1, and GABA, and a decrease in hippocampal ASL perfusion, as well as hippocampal neuron damage.

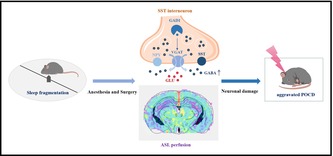

## 1 INTRODUCTION

Postoperative cognitive dysfunction (POCD) is a prevalent neurocognitive complication among elderly patients undergoing anesthesia and surgery, classified under perioperative neurocognitive disorders (Evered et al., 2018). Clinical evidence indicates that aging, anesthesia, and surgery are significant risk factors for POCD (Evered & Silbert, 2018), yet the pathophysiological mechanisms underlying POCD remain inadequately understood (Lin et al., 2020). Notably, preoperative sleep disturbances, particularly sleep fragmentation (SF), are widespread among elderly patients (O'Gara et al., 2021; Stewart & Arora, 2022). SF, a symptom associated with various clinical conditions such as obstructive sleep apnea (OSA) (Carvalho et al., 2023), posttraumatic stress disorder (Lipinska & Thomas, 2019), chronic pain (Mundt et al., 2018), and periodic limb movements (Trenkwalder & Paulus, 2010), is characterized by frequent sleep–wake transitions, leading to reduced sleep depth and quality (Tartar et al., 2006). In elderly patients with OSA, POCD may be a secondary outcome of recurrent SF prior to surgery (Wu, Pu, et al., 2023), and SF has been linked to an increased risk of POCD (Li et al., 2023). Preclinical data suggest that elderly patients are especially vulnerable to SF (Li et al., 2022), and factors such as environmental, medical, and patient‐specific variables may further fragment sleep before surgery (Stewart & Arora, 2022). However, the extent to which preoperative SF heightens susceptibility to POCD in elderly patients undergoing anesthesia and surgery, along with the underlying mechanisms, remains unclear.

Extensive research has elucidated various pathogenetic pathways and preventive measures for POCD (Evered & Silbert, 2018; Lin et al., 2020), with significant success in reducing its incidence (Vutskits & Xie, 2016; Zhang et al., 2023). Perioperative sleep disturbances and POCD share several neurobiological mechanisms, including neuroinflammation, neurotransmitter imbalances, and synaptic dysfunction (Lin et al., 2020; Wang et al., 2021). Unfortunately, studies exploring the role of SF in POCD in the elderly are scarce, with existing research primarily focusing on hippocampal neuroinflammation, yielding conflicting results that fail to fully explain the potential mechanisms of SF in POCD (Lu et al., 2020; Vacas et al., 2017). Recent evidence suggests that normal hippocampal neurotransmitter transmission, particularly involving gamma‐aminobutyric acid (GABA) and glutamate (Glu), is closely tied to the functional state of the aging brain (Castro et al., 2022). SF, surgical stress, and inhalation anesthesia are known to disrupt the hippocampal GABAergic and glutamatergic neurotransmitter systems (Li et al., 2023; Lian et al., 2021; Yu et al., 2019), thereby affecting neuronal excitability and intracellular signaling pathways, which can lead to POCD in aged animal models (Lin et al., 2020). Notably, neurotransmitter disturbances may serve as early indicators of cognitive impairment, preceding observable changes in brain structure and morphology (Song et al., 2021). Additionally, GABAergic and glutamatergic neurotransmitters regulate hippocampal perfusion (Cho et al., 2021), which is a key determinant of hippocampal‐dependent cognitive function (Duan et al., 2020).

In memory research, neuroscientists typically focus on excitatory neurons that release Glu. Within the hippocampus, an essential brain region for memory, these excitatory neurons are predominantly pyramidal neurons, which are critical for the stability of spatial memory (Luo et al., 2019). However, another important group of hippocampal neurons, somatostatin (SST)‐expressing inhibitory interneurons, also play a vital role in spatial memory by releasing GABA to synergistically regulate the function of pyramidal neurons (Racine et al., 2021). Recent studies indicate that hippocampal SST interneurons contribute to the reduction in overall activity of hippocampal pyramidal neurons induced by sleep deprivation, disrupting the consolidation of hippocampal‐dependent memory (Delorme et al., 2021). Moreover, SST interneurons and glutamatergic excitatory neurons are key targets of isoflurane anesthesia, potentially playing a role in the POCD process induced by isoflurane/surgery.

Sleep disturbances, including SF, can induce metabolic disruptions in hippocampal GABA and Glu neurotransmitters (Li et al., 2023; Liang et al., 2023) and alter regional cerebral blood flow (rCBF) within the hippocampus (L'Heureux et al., 2021; Zhou et al., 2019), potentially exacerbating cognitive dysfunction following anesthesia/surgery in aged mice. Therefore, this study aimed to explore the impact of preoperative SF on hippocampal SST interneurons, GABAergic and glutamatergic neurotransmitter levels, and perfusion in a POCD model induced by anesthesia/surgery in aged male mice. We hypothesized that preoperative SF would intensify anesthesia/surgery‐induced cognitive impairment by altering the number of SST interneurons in the hippocampus, accompanied by imbalances in GABAergic and glutamatergic metabolism, rCBF reduction, and neuronal damage.

## 2 MATERIALS AND METHODS

### 2.1 Animals

The animal experiments were conducted in accordance with the guidelines of the Ethics Committee for Experimental Animal Welfare of Tianjin Medical University General Hospital and adhered to the National Institutes of Health Guide for the Care and Use of Laboratory Animals. Based on previous research (Dong et al., 2016), 18‐month‐old male mice were selected to represent the aging state, as this age in C57BL/6J mice corresponds to the onset of the human aging process. Eighteen‐month‐old male C57BL/6J mice were procured from Huafukang Biotechnology Ltd. (Beijing, China). The mice were housed in a controlled environment with an ambient temperature of 22 ± 1°C, 40% to 50% humidity, and a 12:12‐h light/dark cycle (lights on at 7:00 a.m., lights off at 7:00 p.m.), with unrestricted access to purified food (Huafukang Biotechnology Ltd., Beijing, China) and water. The animals were acclimated to these conditions for 1 week.

### 2.2 Experimental design

**FIGURE 1:**
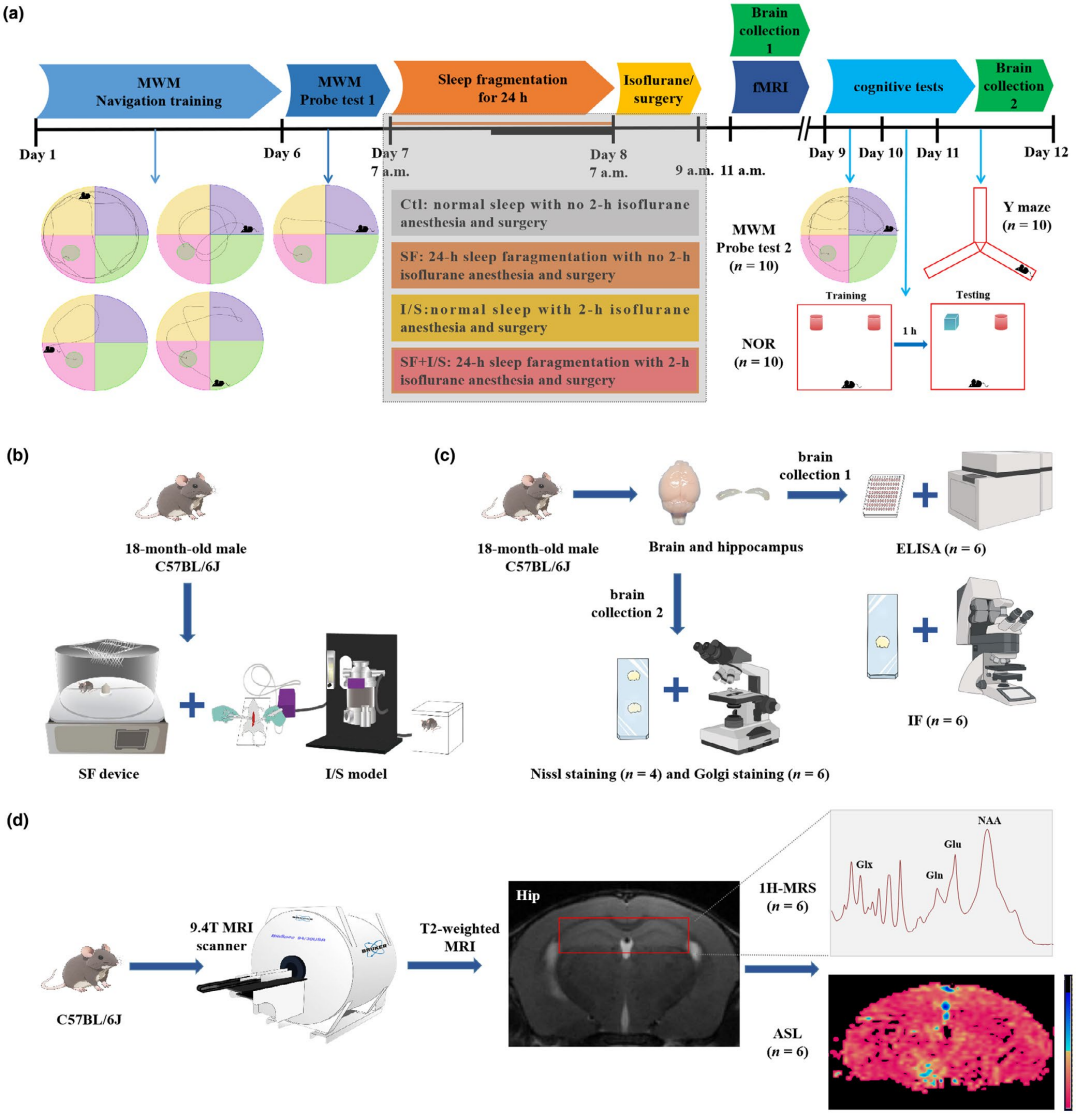
Schematic diagram of experimental design. (a) The timeline of the experiments, including sleep fragmentation (SF) modeling, isoflurane anesthesia and surgery (I/S) modeling, experimental grouping, in vitro assays, in vivo MRI, and behavioral testing of POCD, including the MWM, NOR, and Y maze tests with 10 mice per group. The experiment was divided into four groups: Clt, SF, I/S and SF + I/S. (b) Establishing preoperative 24‐h SF and I/S models. (c) In vitro assays: The brain and hippocampus were collected to ELISA and IF, as well as the Nissl and Golgi staining. Each assay item has 6 samples per group. (d) In vivo MRI: A 9.4 T small animal MRI for the 1H‐MRS and ASL detection in the hippocampus with 6 mice per group. 1H‐MRS, Proton magnetic resonance spectroscopy; ASL, Arterial spin labeling; ELISA, Enzyme‐linked immunosorbent assay; I/S, Isoflurane and surgery; IF, Immunofluorescence; MRI, Magnetic resonance imaging; MWM, Morris water maze; NOR, Novel object recognition; POCD, Postoperative cognitive dysfunction; SF, Sleep fragmentation.

According to the experimental design, the mice were randomly assigned to one of four groups (Day 7–8): (i) control group (Ctl), (ii) SF group, (iii) isoflurane anesthesia and surgery group (I/S), and (iv) SF combined with isoflurane anesthesia and surgery group (SF + I/S) and subjected to three independent procedures (Figure 1). In part 1, the effect of preoperative SF on POCD susceptibility in the four groups (*n* = 10) was assessed. Mice with adequate Morris water maze (MWM) training (Day 1–6) were exposed to 24‐h SF (Day 7) prior to anesthesia and surgery (Day 8), followed by the MWM probe test, novel object recognition (NOR) test, and Y maze test from Day 9 to Day 11. In part 2, changes in hippocampal glutamatergic metabolism and perfusion volume were evaluated in vivo using a 9.4 T animal magnetic resonance imaging (MRI) scanner on Day 8, with proton magnetic resonance spectroscopy (1H‐MRS) and arterial spin labeling (ASL) applied to the four groups (*n* = 6). In part 3, mice were euthanized with an intraperitoneal injection of 150 mg/kg pentobarbital sodium (Huaxia Chemical Reagent Co., Ltd., Sichuan, China). Four in vitro molecular biological assays were conducted on the four groups (*n* = 6), including enzyme‐linked immunosorbent assay (ELISA) and immunofluorescence (IF) to detect the number of SST interneurons and GABAergic markers in the hippocampus from brain collection 1 (Day 8). Additionally, Nissl and Golgi staining were employed to observe changes in Nissl bodies and dendritic spine density in the hippocampal CA1 region from brain collection 2 (Day 11).

### 2.3 SF procedure

The SF model was established using the sleep deprivation rod method (Wu, Li, et al., 2023). Mice were placed in a sleep deprivation apparatus (Beijing Zhongshi Dichuang Technology Development Co., LTD), consisting of a cylindrical container with a diameter of 30 cm and a height of 35 cm, equipped with a rotating rod at the bottom. The device maintained a regular light (lights on at 7:00 a.m.)/dark (lights off at 7:00 p.m.) cycle, with unrestricted access to food and water. The rotating rod was programmed to operate in a 30‐s on/90‐s off cycle. During the 30‐s active phase, the rod rotated periodically, forcing the mice to move or cross the rod, thereby disrupting their sleep. In the 90‐s interval phase, the rod remained stationary, allowing the mice to obtain brief periods of sleep. The SF procedure was conducted from 7:00 a.m. for a duration of 24 h, with a sleep interruption frequency of 30 times per hour. This model simulates the SF commonly observed in OSA (Tartar et al., 2006) and has been validated as an animal model for preoperative SF (Wu, Li, et al., 2023).

### 2.4 POCD model

The right carotid artery exposure surgery under isoflurane anesthesia was employed to induce POCD (Li et al., 2023). Mice were anesthetized with 1.8% isoflurane, and after a minimum of 30 min, a 1.5 cm incision was made along the midline of the neck, with gentle retraction of the soft tissue over the trachea. Careful dissection was performed to expose a 1 cm segment of the right common carotid artery, ensuring the vagus nerve remained intact. The wound was then irrigated and closed with surgical sutures. The procedure, conducted under sterile conditions, lasted approximately 15 min. Postoperatively, animals received a subcutaneous injection of 3 mg/kg bupivacaine for analgesia. The total duration of isoflurane anesthesia was 2 h, a clinically relevant period, during which the animals were kept on a warm pad.

### 2.5 Behavioral tests

#### 2.5.1 MWM

The MWM test was used to evaluate hippocampal‐dependent long‐term spatial memory, comprising three main phases: training, probe test, and memory test (Wadhwa et al., 2017). The testing apparatus consisted of a circular pool (Xinruan Information Technology Ltd., Shanghai, China) with a diameter of 120 cm, a height of 45 cm, and water maintained at 20 ± 2°C. During the training phase (Day 1–5), the pool was divided into four zones (Z1‐4), with the platform placed in the Z4 zone, designated as the target quadrant. Mice were sequentially placed in zones Z1, Z2, Z3, and Z4 for 60 s each, and given four attempts per day to locate the platform. If a mouse failed to find the platform within 60 s, the experimenter guided it to the platform and allowed it to remain there for 15 s. On Day 6, during the probe test, mice were placed in the Z2 zone for 1 min to confirm adequate training, as indicated by locating the platform within 10 s, ensuring the animals were well‐prepared for the grouping procedure. In the memory test phase (Day 9), mice from the four groups were placed in the Z2 zone for 60 s without the hidden platform. The spatial memory test evaluated parameters such as the number of platform crossings and the time spent in the target quadrant.

#### 2.5.2 NOR

The NOR test assessed hippocampal‐dependent short‐term spatial memory through habituation, training, and testing phases (Leger et al., 2013). The test was conducted in a square box (50 × 50 × 40 cm), with a camera installed overhead and connected to the SuperMaze Animal Behavior Video Analysis System (Xinruan Information Technology Ltd., Shanghai, China). After a day of habituation (Day 9), mice were placed in the box with two identical cylindrical objects and allowed to explore for 5 min during the training phase (Day 10). One hour later, one cylindrical object was replaced with a novel rectangular object, and the mice underwent a 5‐min testing phase to assess their recognition of the new object. The exploration time for the novel object and the discrimination index (exploration time for the novel object/total exploration time for both objects) were recorded and analyzed.

#### 2.5.3 Y maze

The Y‐maze test was employed to evaluate hippocampal‐dependent short‐term spatial memory based on the mouse's instinct to explore new environments (Kraeuter et al., 2019). The apparatus consisted of three arms arranged at 120° angles to each other, designated as the start arm, familiar arm, and novel arm. Initially, the mice underwent adaptive training (Day 11), where they were placed at the end of the start arm and allowed to freely explore the start and familiar arms for 10 min. After 1 h, the novel arm was opened, and the mice were repositioned in the start arm to begin the testing phase, during which they could explore all three arms of the maze for 5 min. Video recordings were analyzed to assess parameters such as time spent in the novel arm and the number of entries into the novel arm.

### 2.6 MRI acquisition

In vivo MRI scanning was conducted using a 9.4 T small animal MRI superconducting magnet (BioSpec 94/30 USR; Bruker, Germany). Mice were anesthetized with 2% isoflurane mixed with oxygen and positioned prone on the MRI scanner bed. An animal vital signs monitoring system was employed to maintain the respiratory rate of the mice at 80–100 breaths per minute, while a circulating warm water system at 37°C was used to keep the animals warm. T2‐weighted imaging (T2WI) was acquired using the Rapid Acquisition with Relaxation Enhancement (RARE) sequence with the following parameters: repetition time (TR)/echo time (TE) = 4808/33 ms, field of view (FOV) = 20 × 20 mm^2^, image size = 256 × 256, slice thickness = 0.5 mm, number of averages (NA) = 2, and 50 slices. T2WI was used to locate the hippocampal region for subsequent 1H‐MRS and ASL perfusion MRI.

#### 2.6.1 1H‐MRS

In vivo 1H‐MRS of the hippocampus was performed using a point‐resolved spectroscopy (PRESS) sequence with parameters: TR/TE = 1500/16.5 ms, 256 averages, and a target voxel of interest measuring 4 × 1 × 2 mm^3^. Data were processed semi‐automatically using Bruker ParaVision 360 software to obtain spectral curves, which were then analyzed and quantified using Mestrenova software (V12.0.0, MestreLab Research, Spain). Glutamatergic metabolites in the hippocampus were quantified as ratios of Glu/creatine (Cr), glutamine (Gln)/Cr, and Glu + Gln (Glx)/Cr, using Cr as a standard reference. Additionally, N‐acetyl aspartate (NAA)/Cr was measured as a metabolic marker to reflect the functional integrity of hippocampal neurons.

#### 2.6.2 ASL

In vivo ASL perfusion of the hippocampus was conducted following the 1H‐MRS scan. For ASL imaging, the scanning plane was first aligned with the hippocampal level used in 1H‐MRS, based on T2WI. The Echo Planar Imaging‐Fluid Attenuated Inversion Recovery (EPI‐FLAIR) sequence was employed with the following parameters: TR/TE = 4808/33 ms, FOV = 20 × 20 mm^2^, image size = 128 × 128, and a single slice. ASL perfusion images of the hippocampus were reconstructed using Bruker ParaVision 360 software.

### 2.7 Elisa

The hippocampus was rapidly harvested from mice 2 h postanesthesia and surgery, lysed with radioimmunoprecipitation assay buffer (Abcam, ab156034, UK), homogenized, and centrifuged at 12,000 g for 10 min at 4°C. Following the manufacturer's instructions, levels of SST (MM‐0493 M1), neuropeptide Y (NPY) (MM‐0501 M1), glutamic acid decarboxylase 1 (GAD1) (MM‐46251 M1), vesicular GABA transporter (VGAT) (MM‐47330 M1), GABA (MM‐0442 M1), and Glu (MM‐44113 M1) in the hippocampus were quantified using ELISA kits (Jiangsu Meimian Industrial Co., Ltd., China) by measuring absorbance (OD value) at a wavelength of 450 nm.

#### 2.7.1 Immunofluorescence

The brains were collected following perfusion with normal saline and fixed in 4% paraformaldehyde 24 h at 4°C, embedded in paraffin wax, and sectioned into 10‐μm coronal slices. The hippocampal sections were blocked with goat serum and incubated overnight at 4°C with an Anti‐Mouse SST primary antibody (1:100, Sc‐74,556, Santa Cruz Biotechnology, USA), followed by incubation with Goat Anti‐Mouse IgG H&L (Alexa Fluor® 647) (1:500, ab150115, Abcam, UK) and DAPI (1:500, ab104139, Abcam, UK) for 40 min at room temperature. Fluorescence images of the whole brain were captured using an Olympus BX51 microscope. Regions of interest (ROIs) were set in the CA1, CA3, and DG subregions of the hippocampus using Caseviewer 2.4 software (3DHISTECH Ltd.) and fluorescence images of ROIs were captured at 40× microscope for quantification. The number of SST‐positive cells in the CA1, CA3, and DG of the hippocampus was quantified using ImageJ software (National Institutes of Health, Bethesda, MD, USA).

### 2.8 Nissl staining

At the conclusion of behavioral tests, mice were perfused transcardially, and their brains were fixed in 4% paraformaldehyde for 24 h before being embedded in paraffin. The paraffin‐embedded brain samples were sectioned into 6‐μm coronal slices using a microtome (HistoCore MULTICUT, Leica, Germany). The hippocampal sections were deparaffinized with xylene, rehydrated through a graded ethanol series, and rinsed twice with PBS for 5 min each. Nissl staining solution (G1434, Beijing Solarbio Science & Technology Co., Ltd., China) was applied uniformly to the hippocampal sections. After washing twice with PBS (5 min each), the sections were dehydrated with anhydrous alcohol for 15 min, cleared with xylene for 15 min, and mounted with neutral gum. Neurons and Nissl bodies in the pyramidal cell layer of the CA1 region of the hippocampus were observed under a high‐power microscope (40×), and images were captured for subsequent analysis.

### 2.9 Golgi staining

At the conclusion of behavioral tests, mice were euthanized to obtain brain tissues, which were then processed for Golgi‐Cox staining using a Rapid Golgi Stain Kit (PK401, FD Neuro‐Technologies, Ellicott City, MD, USA). Briefly, at room temperature and shielded from light, the brain samples were immersed in a mixture of solutions A and B for 2 weeks, followed by immersion in solution C for 1 week. The brains were then sectioned into 100‐μm coronal slices using a microtome (VT1200S, Leica, Germany) and mounted on glass slides. Neuronal dendritic spines in the hippocampal CA1 region were observed using a 100× oil immersion objective under a vertical optical microscope (Olympus), and the number of dendritic spines was quantified using ImageJ software.

### 2.10 Statistical analysis

Statistical analyses of the experimental data were conducted using GraphPad Prism 9.5 (GraphPad Software, San Diego, USA). Continuous variables were expressed as mean ± standard deviation (SD). The count data of number of platform crossings in the MWM and entries into the novel arm in the Y maze were analyzed with the nonparametric Kruskal–Wallis test followed by Dunn's multiple comparisons. The other data of multiple group comparisons were performed using one‐way analysis of variance (ANOVA) followed by Tukey's post hoc test. Post hoc effect sizes were calculated with G*Power, assuming 80% power and a type I error rate of 0.05 post hoc test (Faul et al., 2007). A *p*‐value of <0.05 was considered statistically significant.

## 3 RESULTS

### 3.1 Preoperative 24‐h SF aggravated POCD induced by isoflurane anesthesia/ surgery in aged mice

**FIGURE 2:**
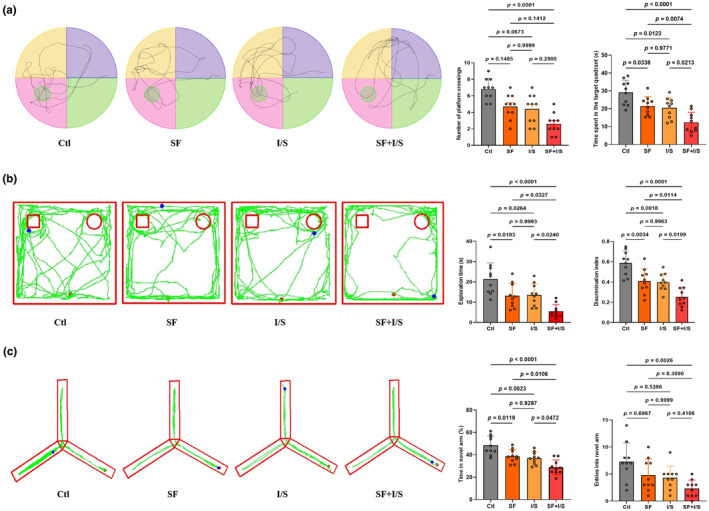
Preoperative 24‐h SF aggravated POCD induced by the anesthesia/surgery in aged mice. (a) Representative trajectory plot and statistical analysis of the number of platform crossings (Kruskal–Wallis test with Dunn's multiple comparisons) and time spent in the target quadrant (one‐way ANOVA with Tukey's post hoc test) in the MWM test. (b) Representative trajectory plot and statistical analysis of exploration time and discrimination index for novel object in the NOR test (one‐way ANOVA with Tukey's post hoc test). (c) Representative trajectory plot and statistical analysis of time percentage of novel arm (one‐way ANOVA with Tukey's post hoc test) and entries into novel arm (Kruskal–Wallis test with Dunn's multiple comparisons) in the Y maze test. Using G*Power to analyze the data indicated effect size to be 0.55. The data are represented as the mean ± SD with *n* = 10/group.

To investigate whether preoperative 24‐h SF exacerbates anesthesia/surgery‐induced cognitive impairment, a series of sequential behavioral tests were performed. Compared to the control group, the I/S group exhibited impaired cognitive performance in aged mice, as evidenced by the MWM, NOR, and Y maze tests (Figure 2). These results confirm that isoflurane anesthesia combined with surgery successfully induced POCD in aged mice. Compared with the I/S mice, SF + I/S mice showed shorter time spent in the target quadrant (*p =* 0.0213) and no difference in the number of platform crossings (*p =* 0.2900) in the MWM test (Figure 2a). Likewise, in the NOR test, SF + I/S mice showed less exploration time for the novel object (*p* = 0.0240) and a lower discrimination index (*p* = 0.0199) compared with the I/S mice (Figure 2b). SF + I/S mice also showed a decrease in the percentage of novel arm time (*p* = 0.0472) not the entries into the novel arm (*p* = 0.4108) in the Y maze test compared with the I/S mice (Figure 2c). Additionally, no significant differences were observed in the three behavioral tests between the SF and I/S groups.

### 3.2 Preoperative 24‐h SF promoted the anesthesia/surgery‐induced increase of GABAergic neurotransmitters in the hippocampus

**FIGURE 3:**
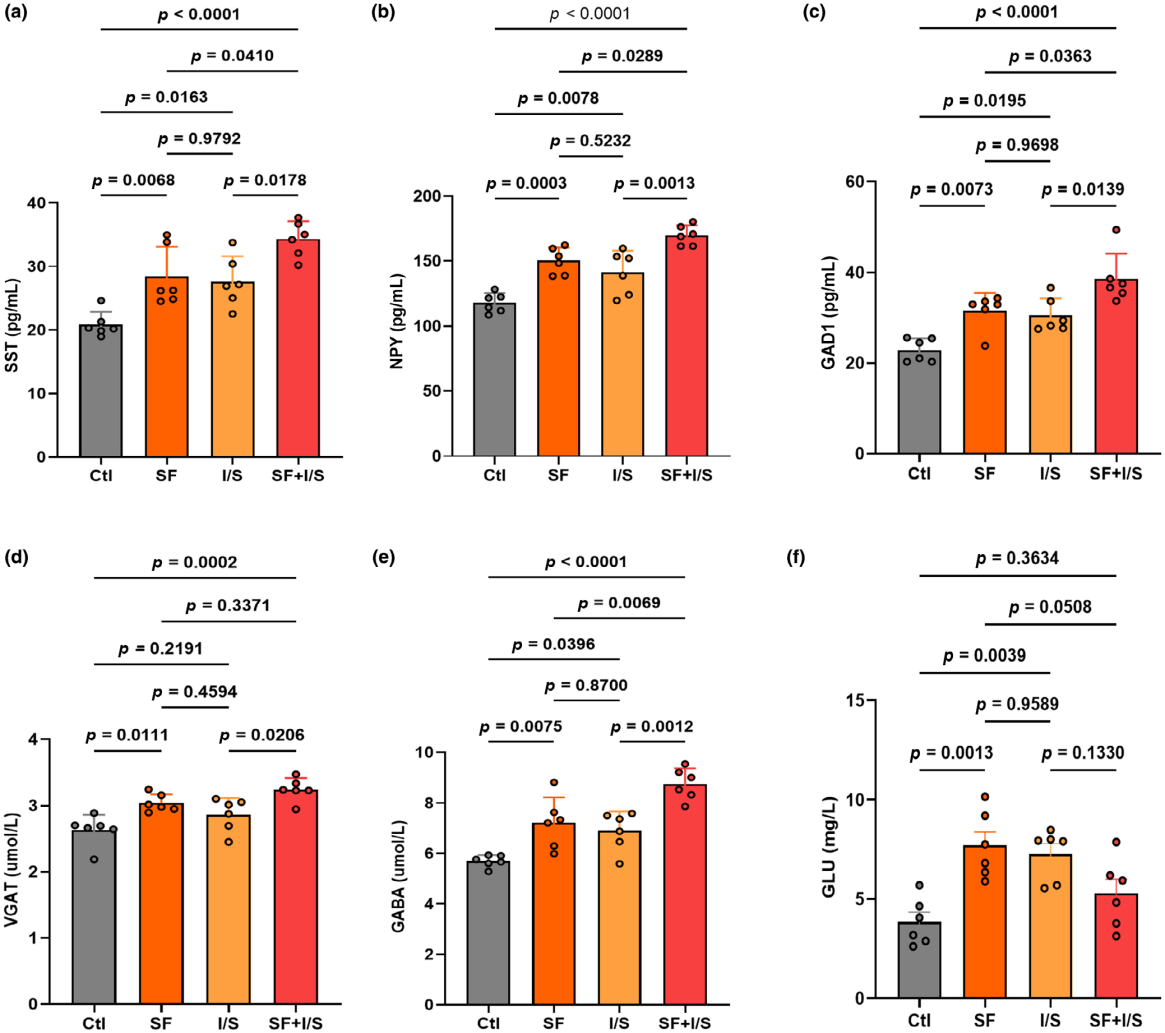
Preoperative 24‐h SF promoted the anesthesia/surgery‐induced increase of GABAergic neurotransmitters in the hippocampus. The SST, NPY, GAD1, VGAT, GABA, and GLU levels in the hippocampus as detected by ELISA. Levels of the SST (a), NPY (b), GAD1 (c), VGAT (d), GABA (e), and GLU (f) in the hippocampus are presented as mean ± SD with *n* = 6/group and were analyzed by one‐way ANOVA with Tukey's post hoc test. Using G*Power to analyze the data indicated effect size to be 0.74.

Given that NPY, GAD1, and VGAT are enriched in SST‐expressing interneurons, which also release GABA, ELISA was performed to quantify these neurotransmitters and GLU levels. The levels of SST (*F* [3, 20] = 14.66, SF vs. Clt: *p* = 0.0068, I/S vs. Clt: *p* = 0.0163, Figure 3a), NPY (*F* [3, 20] = 22.62, SF vs. Clt: *p* = 0.0003, I/S vs. Clt: *p* = 0.0078, Figure 3b), GAD1 (*F* [3, 20] = 14.80, SF vs. Clt: *p* = 0.0073, I/S vs. Clt: *p* = 0.0195, Figure 3c), and GABA (*F* [3, 20] = 18.50, SF vs. Clt: *p* = 0.0075, I/S vs. Clt: *p* = 0.0396, Figure 3e) in the hippocampus of SF and I/S groups were higher than those of the Clt group. However, in the level of VGAT (*F* [3, 20] = 9.859, SF vs. Clt: *p* = 0.0111, I/S vs. Clt: *p* = 0.2191, Figure 3d), SF mice not I/S mice had a significant increase in the parameter compared with the Clt mice. Compared with the I/S mice, SF + I/S mice exhibited higher levels of NPY (*p* = 0.0013), GAD1 (*p* = 0.0139), VGAT (*p* = 0.0206), and GABA (*p* = 0.0012). When detecting the level of GLU among the groups (*F* [3, 20] = 8.626, SF vs. Clt: *p* = 0.0013, I/S vs. Clt: *p* = 0.0039, Figure 3f), we found that SF and I/S increased the parameter compared with the Clt (*p* = 0.9589) and that SF + I/S decreased the parameter with no difference compared with the SF (*p* = 0.0508) and I/S (*p* = 0.1330). In addition, compared with the Clt mice, the level of GLU in SF + I/S mice increased but had no difference (*p* = 0.3634). Additionally, GLU levels in the SF + I/S group were elevated compared to the Clt group, but this difference was not statistically significant (*p* = 0.3634).

### 3.3 Preoperative 24‐h SF increased the number of SST interneurons in the CA1 region of the hippocampus

**FIGURE 4:**
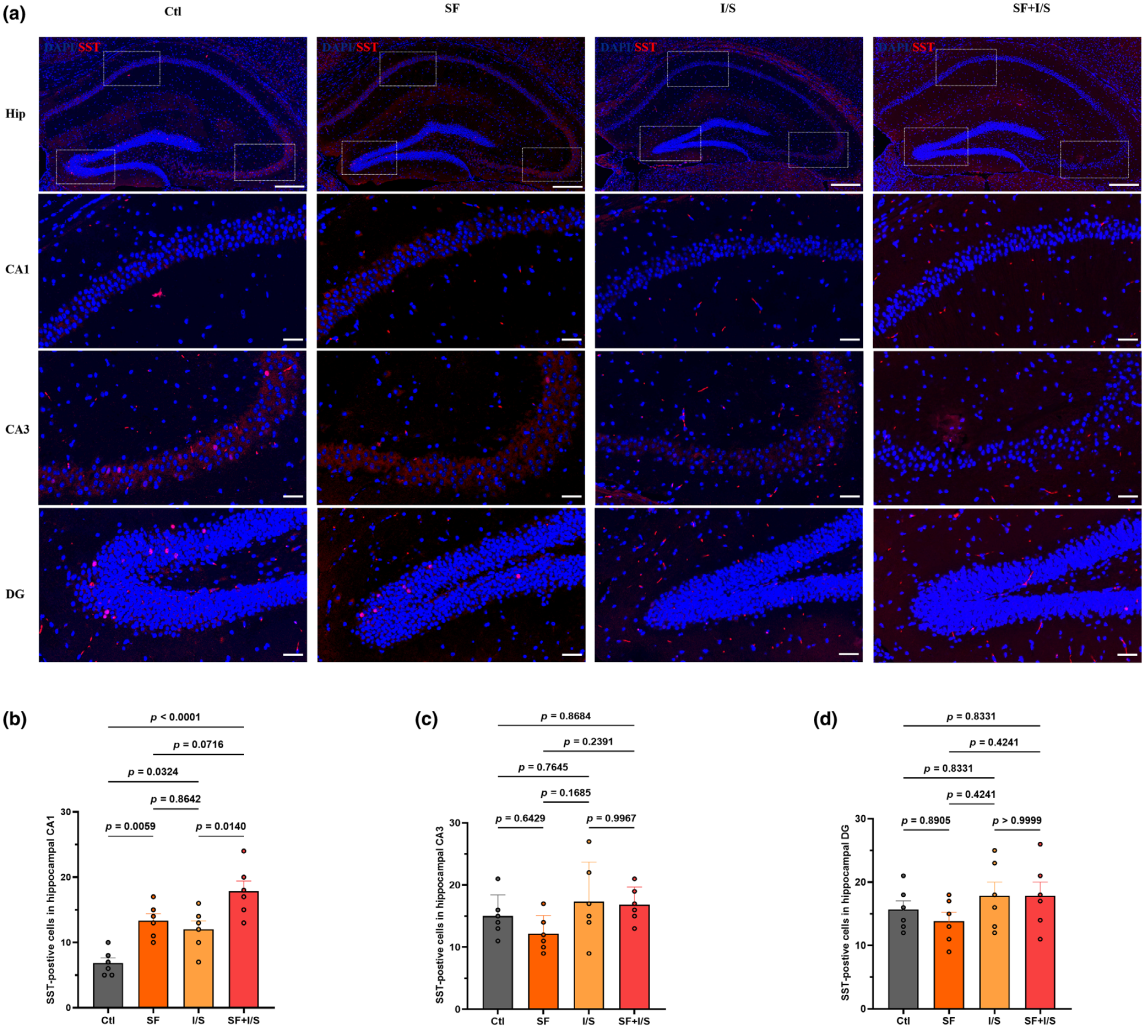
Preoperative 24‐h SF increased the number of SST interneurons in the CA1 region of the hippocampus. (a) Immunofluorescence staining of SST‐positive cells in the hippocampus (scale bar = 100 μm), including the CA1, CA3, and DG regions (scale bar = 20 μm). Quantification of SST‐positive cells in the CA1 (b), CA3 (c), and DG (d) of the hippocampus. G*Power to analyze the data indicated effect size to be 0.74. The data are presented as mean ± SD with *n* = 6/group and were analyzed by one‐way ANOVA with Tukey's post hoc test.

SST interneurons are crucial in sleep disorder‐related cognitive impairment and isoflurane anesthesia. To evaluate their distribution, SST‐specific markers were detected in the CA1, CA3, and DG subregions of the hippocampus using immunofluorescence staining (Figure 4a). In the CA1 (*F* [3, 20] = 13.90, *p* < 0.0001, Figure 4b), the SF, I/S, and SF + I/S groups showed more SST‐positive cells compared with the Clt group (SF vs. Clt: *p* = 0.0059, I/S vs. Clt: *p* = 0.0324, SF + I/S: *p* < 0.0001), SF + I/S led to a significant increase in SST interneuron activation compared with the I/S group (*p* = 0.0140), and there was no difference in the number of SST‐positive cells between the SF and I/S groups (*p* = 0.8642). In contrast, no significant differences in SST‐positive cells were found among the four groups in the CA3 (*F* [3, 20] = 1.911, *p* = 0.1603, Figure 4c) and DG (*F* [3, 20] = 1.131, *p* = 0.3606, Figure 4d) regions.

### 3.4 Preoperative 24‐h SF did not accelerate the anesthesia/surgery‐induced increase of glutamatergic neurotransmitters in the hippocampus

**FIGURE 5:**
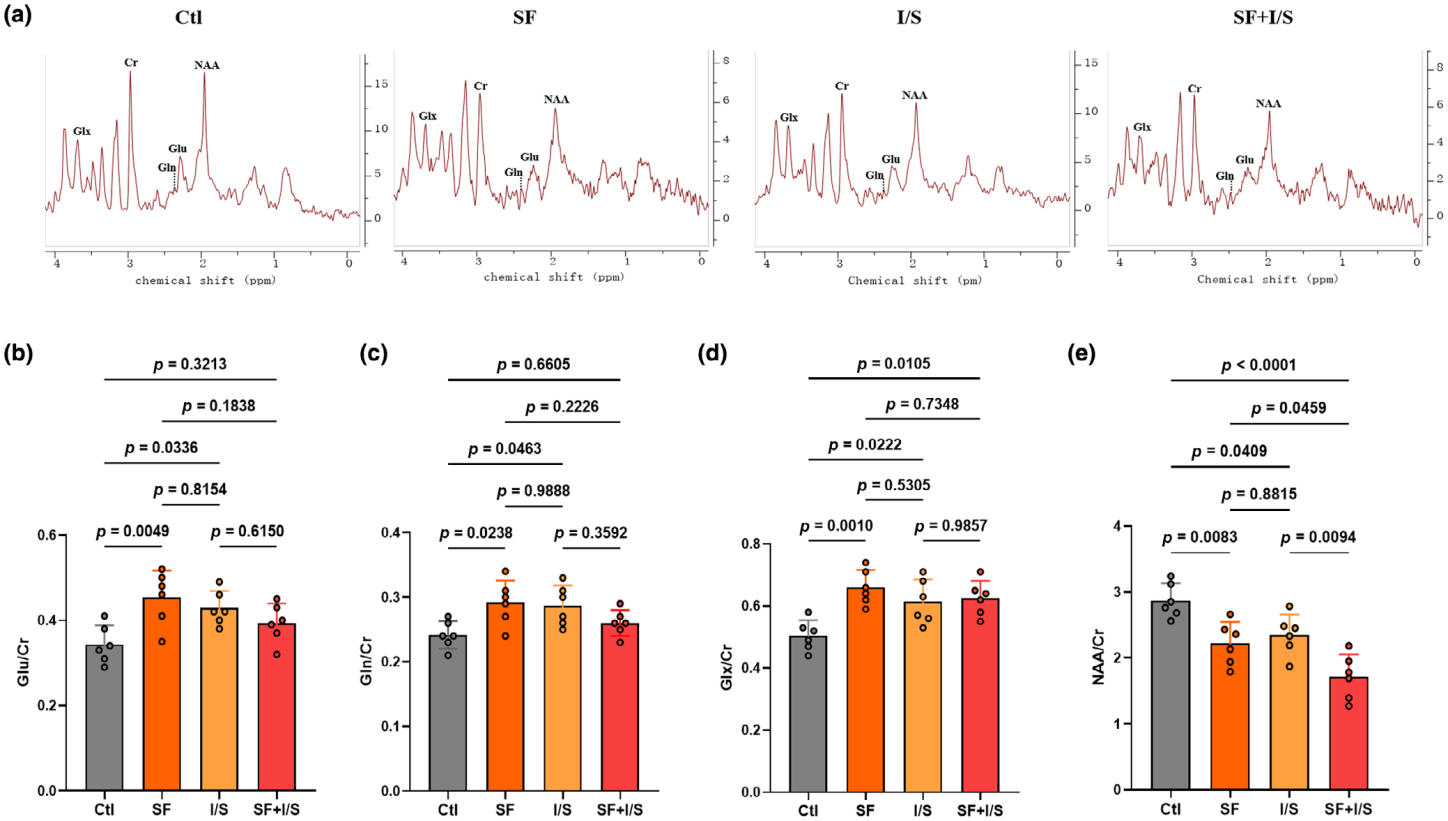
Preoperative 24‐h SF did not accelerate the anesthesia/surgery‐induced increase of glutamatergic neurotransmitters in the hippocampus. (a) Representative metabolite spectrum of Glu, Gln, Glx, NAA, and Cr in the hippocampus as detected by 1H‐MRS. Changes in the ratios of Glu/Cr (b), Gln/Cr (c), Glx/Cr (d), and NAA/Cr (e). Using G*Power to analyze the data indicated effect size to be 0.74. The data are presented as mean ± SD with *n* = 6/group and were analyzed by one‐way ANOVA with Tukey's post hoc test.

To determine if preoperative SF influences isoflurane anesthesia/surgery‐induced changes in glutamatergic metabolism, spectra of Glu, Gln, and Glx in the hippocampal region were examined using in vivo 1H‐MRS, along with NAA, a recognized marker of neuronal integrity (Figure 5a). Both SF and I/S increased the ratio of Glu/Cr (*F* [3, 20] = 5.626, SF vs. Clt: *p* = 0.0049, I/S vs. Clt: *p* = 0.0336, Figure 5b), Gln/Cr (*F* [3, 20] = 4.371, SF vs. Clt: *p* = 0.0238, I/S vs. Clt: *p* = 0.0463, Figure 5c), and Glx/Cr (*F* [3, 20] = 7.734, SF vs. Clt: *p* = 0.0010, I/S vs. Clt: *p* = 0.0222, Figure 5d) compared with the Clt. There were no differences in the ratio of Glu/Cr (*p* = 0.8154), Gln/Cr (*p* = 0.9888), and Glx/Cr (*p* = 0.5305) between the SF and I/S mice. However, compared with the I/S group, SF + I/S decreased the ratio of Glu/Cr (*p* = 0.6150) and Gln/Cr (*p* = 0.3592) with no difference. In addition, in the ratio of NAA/Cr (*F* [3, 20] = 14.15, *p* < 0.0001, Figure 5e), SF, I/S, and SF + I/S decreased compared with the Clt (SF vs. Clt: *p* = 0.0083, I/S vs. Clt: *p* = 0.0409, SF + I/S: *p* < 0.0001). Compared with the I/S, SF + I/S diminished the ratio of NAA/Cr in the hippocampus (*p* = 0.0094). There was no difference in the ratio of NAA/Cr between the SF and I/S mice (*p* = 0.8815).

### 3.5 Preoperative 24‐h SF augmented the anesthesia/surgery‐induced reduction of hippocampal perfusion

**FIGURE 6:**
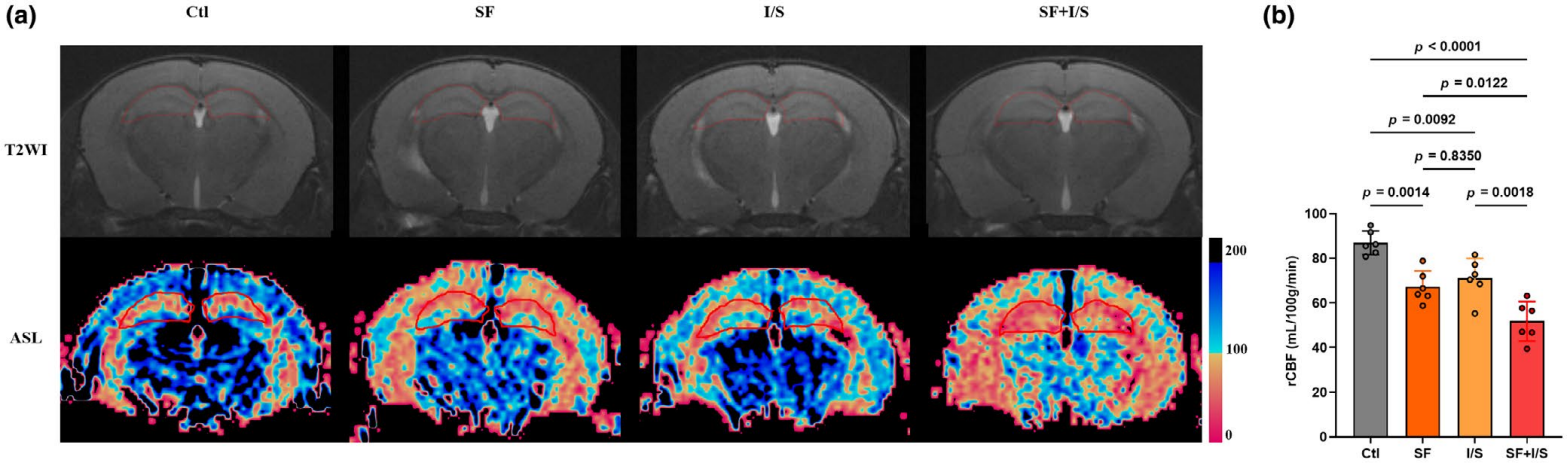
Preoperative 24‐h SF augmented the anesthesia/surgery‐induced reduction of hippocampal perfusion. (a) Representative T2WI and ASL images of the hippocampus. (b) Changes in rCBF in the hippocampus. Using G*Power to analyze the data indicated effect size to be 0.74. The data are presented as mean ± SD and were analyzed by one‐way ANOVA with Tukey's post hoc test.

Hippocampal perfusion is fundamental to the functioning and maintenance of the hippocampus, which plays a role in sleep regulation and hippocampal‐dependent cognitive processing. Hippocampal perfusion was assessed using in vivo MRI, and ASL heat maps of the hippocampus were generated (Figure 6a). As showed in Figure 6b (*F* [3, 20] = 20.94, *p* < 0.0001), SF, I/S, and SF + I/S induced a significant decrease in the hippocampal rCBF compared with the Clt (SF vs. Clt: *p* = 0.0014, I/S vs. Clt: *p* = 0.0092, SF + I/S: *p* < 0.0001), SF + I/S led to a further increase in the hippocampal rCBF compared with the I/S (*p* = 0.0018), and there was no difference in the hippocampal rCBF between the SF and I/S mice (*p* = 0.8350).

### 3.6 Preoperative 24‐h SF exacerbated anesthesia/surgery‐induced neuronal damage in the CA1 region of the hippocampus

**FIGURE 7:**
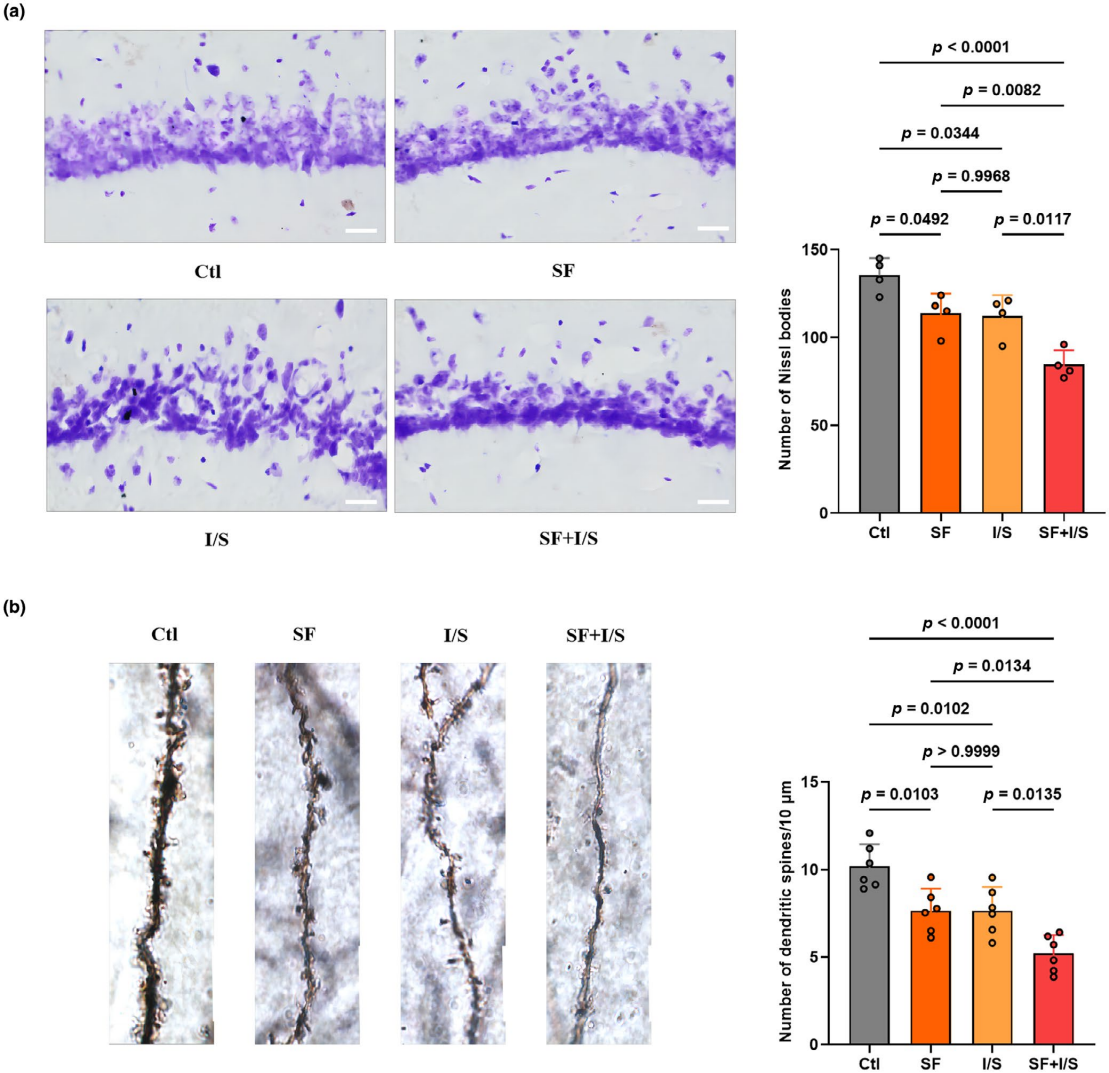
Preoperative 24‐h SF exacerbated anesthesia/surgery‐induced neuronal damage in the CA1 region of the hippocampus. (a) Changes in the number of Nissl bodies in the CA1 region of the hippocampus as detected by Nissl staining. Scale bar = 20 μm, *n* = 4/group, effect size = 0.97. (b) Changes in the number of dendritic spines/10 μm in the CA1 region of the hippocampus as detected by Nissl staining. Scale bar = 5 μm, *n* = 6/group, effect size = 0.74. The data are presented as mean ± SD and were analyzed by one‐way ANOVA with Tukey's post hoc test.

The hippocampal CA1 region plays a critical role in the link between sleep and cognition, where SST interneurons receive diverse neural inputs, including GABAergic and glutamatergic projections, and directly influence pyramidal neurons to sustain spatial memory function. To evaluate neuronal damage in the CA1 region, changes in the number of Nissl bodies and dendritic spine density were observed using Nissl and Golgi staining techniques (Figure 7). In the Nissl staining of hippocampal CA1 neurons (*F* [3, 12] = 16.39, *p* = 0.0002, Figure 7a), SF, I/S, and SF + I/S significantly decreased the number of Nissl bodies compared with the Clt (SF vs. Clt: *p* = 0.0492, I/S vs. Clt: *p* = 0.0344, SF + I/S: *p* < 0.0001), and SF + I/S induced a further decrease in the number of Nissl bodies compared with the I/S (*p* = 0.0117). Likewise, in the Golgi staining of hippocampal CA1 neurons (*F* [3, 20] = 16.12, *p* < 0.0001, Figure 7b), SF, I/S, and SF + I/S showed a significant reduction in the density of dendritic spines compared with the control mice (SF vs. Clt: *p* = 0.0103, I/S vs. Clt: *p* = 0.0102, SF + I/S: *p* < 0.0001), and SF + I/S exhibited a lower density of dendritic spines compared with the I/S mice (*p* = 0.0135). No significant differences were found between the SF and I/S groups regarding the number of Nissl bodies and dendritic spine density.

## 4 DISCUSSION

GABAergic and glutamatergic neurotransmission within the central nervous system is intricately linked to both sleep and cognitive function (Gao et al., 2014; Li et al., 2023). Disruptions in neurotransmitter balance and alterations in rCBF within the hippocampus may unveil the pathological mechanisms underlying various central nervous system disorders. While several studies have independently examined the roles of neurotransmitter imbalances and changes in cerebral blood flow in sleep disturbances—including SF—and POCD, the interaction between these factors has often been overlooked. Given the strong correlation between SF and POCD in elderly patients, this study investigated the impact of preoperative SF on anesthesia/surgery‐induced cognitive decline, as well as its effects on hippocampal neurotransmitters and perfusion. Our findings reveal that preoperative 24‐h SF exacerbates anesthesia/surgery‐induced POCD, evidenced by elevated levels of hippocampal GABAergic metabolites (SST, NPY, GAD1, and GABA), an increased number of SST interneurons in the hippocampal CA1 region, reduced hippocampal rCBF, and neuronal damage, without significant changes in glutamatergic metabolites (Glu, Gln, and Glx).

SF is a symptom associated with numerous clinical conditions, including OSA (Carvalho et al., 2023), pain (Mundt et al., 2018), insomnia, mental health disorders such as depression or anxiety, and external factors like noise, lighting, and medical procedures (Butris et al., 2023). Increasing evidence links OSA with postoperative neurocognitive disorders, including postoperative delirium and POCD, as well as conditions like depression, Alzheimer's disease, and dementia in older adults (Kerner & Roose, 2016). However, the specific relationship between OSA—particularly the SF pattern it induces—and POCD remains underexplored. Given the broad impact of SF, OSA‐induced SF may heighten the susceptibility of patients with OSA to neurocognitive impairment following anesthesia and surgery (Wu, Pu, et al., 2023). The effect of SF on the incidence and severity of postoperative delirium and POCD remains unresolved, with studies reporting conflicting results (Devinney et al., 2022). Some evidence suggests that OSA increases the incidence of POCD in elderly patients undergoing joint replacement (Wu et al., 2022), while other studies show unexpected improvements in memory after surgery in individuals at high risk for OSA, based on the STOP‐BANG score (Wagner et al., 2018). Several confounding factors that may complicate the assessment of OSA's relationship with POCD should be considered, including postoperative pain, opioid use, sleep disturbances, nutritional deficiencies, surgery, and mental status. Furthermore, SF in patients with OSA may worsen after anesthesia and surgery (Tamisier et al., 2018). The precise impact of SF on the risk of postoperative delirium and POCD remains unclear. Our study provides preclinical evidence that SF exacerbates POCD in an aged animal model. The perioperative period is particularly vulnerable to acute sleep disturbances and neurocognitive disorders in the elderly, influenced by medical, environmental, and patient‐specific factors (O'Gara et al., 2021; Wang et al., 2021). A meta‐analysis has shown that sleep disturbances, including SF, increase the risk of perioperative neurocognitive disorders by 4.59‐fold (Fadayomi et al., 2018). Research indicates that postoperative SF increases the risk of POCD in elderly patients (Li et al., 2023; Lu et al., 2020). Notably, elderly patients often experience fragmented sleep before surgery, with reports suggesting a prevalence of over 50% in preoperative elderly patients due to concerns about surgery, adaptation difficulties to the hospital environment, and disease‐related pain (Stewart & Arora, 2022; Wang et al., 2021). However, research on the correlation between preoperative SF and POCD in aged animal models is limited. A previous study (Vacas et al., 2017) found that while SF and surgery independently impaired memory in 12‐ to 14‐week‐old mice, preoperative 24‐h SF significantly increased surgery‐induced hippocampal neuroinflammation without further cognitive decline. Since age is an independent risk factor for POCD (Evered & Silbert, 2018), aging may increase the vulnerability to cognitive decline induced by anesthesia and surgery in the context of SF. In this study, the SF + I/S model in 18‐month‐old mice exhibited more severe POCD compared to the SF or I/S models alone. Although neuroinflammation is a prominent research focus in exploring potential mechanisms of sleep disturbances, including SF, associated with POCD (Cheng et al., 2022), it does not seem to fully explain the pathological process, especially given conflicting findings (Lin et al., 2020). Interestingly, neurotransmitter disorders appear to play a critical sentinel role in age‐related neurocognitive diseases induced by sleep disturbances, anesthesia, and surgical stress, meriting further investigation.

Inhibitory and excitatory neurotransmitters form the biological foundation for brain functions such as sleep–wake regulation, learning, and memory (Brown et al., 2012; Katsuki et al., 2022), and their imbalance may underlie the pathogenesis of neuropsychiatric disorders (Gao et al., 2014). GABAergic inhibitory interneurons and their principal neurotransmitter, GABA, play a pivotal role in regulating anesthesia and sleep‐related cognitive functions (Delorme et al., 2021; Speigel et al., 2022). Recent studies suggest that SST interneurons, rather than parvalbumin interneurons, are the primary targets of isoflurane anesthesia (Speigel et al., 2022; Speigel & Hemmings Jr., 2021). Moreover, sleep disturbances may impair memory consolidation by activating hippocampal SST interneurons, which function as inhibitory gates (Delorme et al., 2021). SST interneurons in the hippocampus inhibit neighboring pyramidal cells and parvalbumin interneurons (Pelkey et al., 2017). Notably, SST interneurons in the hippocampal CA1 region modulate the overall hippocampal network during memory acquisition, with their numbers directly influencing spatial memory (Brzdak et al., 2023; Stefanelli et al., 2016). In this study, preoperative 24‐h SF combined with anesthesia/surgery significantly increased the number of SST interneurons and GABA release throughout the hippocampus, particularly in the CA1 region, compared to SF or anesthesia/surgery alone. These findings suggest that preoperative SF amplifies the effects of isoflurane anesthesia, leading to the increase in the number of SST interneurons, which release excess GABA and contribute to the exacerbation of POCD. Additionally, the potentiating effects of preoperative SF and anesthesia/surgery on the release of glutamatergic neurotransmitters were largely counteracted by the abnormal enhancement of anesthetic potency when both were combined, resulting in a diminished excitatory response to GABAergic neurotransmission. Recent studies have indicated that isoflurane inhalation also affects Glu‐mediated neurotransmission through various mechanisms (Speigel & Hemmings Jr., 2021). Isoflurane not only inhibits Glu release at the presynaptic membrane but also suppresses excitatory postsynaptic potentials mediated by N‐methyl‐D‐aspartic acid receptors (Dong et al., 2013). Glutamatergic neurotransmitters are critical for excitatory synaptic transmission between hippocampal SST interneurons and pyramidal cells, as well as between pyramidal cells, and play a vital role in hippocampal‐dependent sleep and cognition (Brzdak et al., 2023; Delorme et al., 2021; Urban‐Ciecko et al., 2018). Furthermore, acute sleep disturbances and surgical stress have been shown to alter hippocampal glutamatergic neurotransmission (Li et al., 2023), along with increased Glu release from excitatory neurons (Zhang et al., 2022). However, the combined effects of isoflurane anesthesia and surgery on glutamatergic neurotransmitters during sleep disturbances are complex. Our previous study confirmed that postoperative 24‐h SF enhances the release of hippocampal glutamatergic neurotransmitters in aged mice models subjected to isoflurane anesthesia and surgery, contributing to the POCD process (Li et al., 2023). In this study, GABAergic neurotransmission, mediated by SST interneurons, appears to play a dominant role in the exacerbation of POCD induced by preoperative 24‐h SF. Thus, perioperative SF may contribute to POCD through distinct neurometabolic mechanisms, warranting further investigation.

Neuroimaging evidence has demonstrated significant alterations in hippocampal perfusion following acute sleep deprivation (Zhou et al., 2019), indicating that sleep loss negatively impacts the intrinsic functional structure of the hippocampus, a critical region for sleep and cognition. Unlike total sleep deprivation, the SF protocol used in this study simulates disrupted sleep without complete loss, yet it similarly leads to reduced rCBF in both the left and right hippocampus, as shown by ASL perfusion results (Elvsashagen et al., 2019; Zhou et al., 2019). This suggests that even partial sleep disruption can have detrimental effects on hippocampal perfusion. Furthermore, our data indicate that preoperative SF exacerbates hippocampal hypoperfusion following anesthesia and surgery. Functional imaging studies have highlighted the importance of coordination between glutamatergic excitatory neurons and GABAergic inhibitory interneurons in regulating neuronal firing rates, which is relevant in functional magnetic resonance imaging (Donahue et al., 2010; Gao et al., 2014; Krause et al., 2014). Clinical imaging data show that GABA concentrations are strongly correlated with ASL signals, and that GABA is more influential than Glu in regulating rCBF (Krause et al., 2014). In this study, elevated GABA concentrations in the hippocampus were associated with reduced hippocampal perfusion. While rCBF differences related to sleep disturbances suggest their potential as a neural marker with moderate sensitivity and specificity, the direction of the correlation between GABA concentrations and ASL perfusion varies across different brain regions. For instance, 1H‐MRS and ASL imaging data from healthy individuals show a strong negative correlation between GABA levels in the anterior cingulate and whole‐brain CBF (*r* = −0.91, *p* = 0.0015) (Krause et al., 2014), whereas baseline GABA concentrations positively correlate with CBF‐weighted ASL signals (*r* = 0.65, *p* = 0.02) (Donahue et al., 2010). rCBF is essential for cognitive function, serving as a nutrient provider to the hippocampus, a key functional brain region (Duan et al., 2020; Wei et al., 2020). Recent studies suggest that resting CBF and major neurometabolites (GABA and Glu) are closely associated with age‐related cognitive decline (Krishnamurthy et al., 2022), aligning with the primary findings of this study.

This study has certain limitations. To assess the effect of preoperative SF on anesthesia/surgery‐induced POCD, a combined SF and POCD modeling approach was employed. However, this “double hit” model complicates the analysis of SF's causal role in POCD, introducing confounding factors. The animal model of SF used in this study does not fully replicate clinical features, which is a common limitation in preclinical studies and a notable weakness of this research. Additionally, the POCD model, which combines long‐term inhalation anesthesia with short‐term surgical procedures, may not entirely mimic clinical conditions, making it challenging to determine whether inhalation anesthesia or surgical trauma is the primary factor in inducing POCD. Currently, propofol, a noninhalation anesthetic, is widely used and has been shown to have neuroprotective effects (Mardini et al., 2017). However, high doses and prolonged use of propofol also pose a risk of cognitive impairment (Gonzales et al., 2015; Kim et al., 2021). The potential additional effects of preoperative sleep disorders, including SF, on cognitive impairment following propofol anesthesia and surgery remain unexplored and warrant further investigation. The SF protocol was initially designed to mimic sleep characteristics of clinical sleep disorders such as OSA. However, whether it accurately reflects the clinical symptoms of patients experiencing acute SF without obstructive sleep apnea before surgery is still unclear and lacks clinical validation. In the analysis of behavioral tests, due to a lack of multiplicity correction for 6 measures, the *p*‐values reported are probably simultaneously too conservative and not conservative enough. Whether these effects cancel out is not clear and not likely.

## 5 CONCLUSIONS

In summary, this preclinical study confirms that preoperative 24‐h SF exacerbates POCD induced by isoflurane anesthesia and surgery. This is evidenced by an increased number of SST interneurons in the hippocampal CA1 region, elevated levels of hippocampal GABAergic neurotransmitters, reduced rCBF in the hippocampus, and neuronal damage in hippocampal CA1.

## AUTHOR CONTRIBUTIONS

Yun Li conceived the study, supervised the work, wrote the manuscript, and analyzed data. Jiafeng Yu and Yize Li conducted experiments and revised the manuscript. Ningzhi Yang and Siwen Long performed experiments and analyzed data. Lina Zhao and Yonghao Yu designed experiments, supervised the work, and edited the manuscript. All authors have read and approved this manuscript.

## FUNDING INFORMATION

This work was supported by the National Natural Science Foundation of China (82072150, 82071243), Scientific Research Project of Tianjin Municipal Education Commission (2022KJ232), General Project of Tianjin Natural Science Foundation (20JCYBJC00460), and Tianjin key Medical Discipline (Specialty) Construction Project (TJYXZDXK‐036A).

## CONFLICT OF INTEREST STATEMENT

The authors declare no competing interests.

## ETHICS STATEMENT

All animal procedures were approved by the Experimental Animal Welfare Ethics Committee of Tianjin Medical University General Hospital (protocol IRB2023‐DWFL‐391).

## Data Availability

The data in the article are available from the corresponding author upon reasonable request.
